# Familial Hypercholesterolemia: A Comprehensive Review of Advances in Treatment Strategies and the Role of Patient Beliefs

**DOI:** 10.7759/cureus.78032

**Published:** 2025-01-26

**Authors:** Hadi A Bakour, Jumana Hussain Timraz, Bushra Wadi Bin Saddiq, Nourah A Alghamdi, Husna Irfan Thalib, Maryam Alyarimi, Ibraheem Ali Algarni

**Affiliations:** 1 General Medicine and Surgery, Batterjee Medical College, Jeddah, SAU; 2 College of Medicine and Surgery, Fakeeh College of Medical Sciences, Jeddah, SAU; 3 Internal Medicine (Endocrinology), My Clinic, Jeddah, SAU; 4 Family and Community Medicine, Rabigh Faculty of Medicine, King Abdulaziz University, Jeddah, SAU

**Keywords:** advances in treatment, familial hypercholesterolemia, hypercholesterolemia, patient beliefs, treatment choices

## Abstract

Familial hypercholesterolemia (FH) constitutes the most common inherited lipid disorder caused by mutations in any of the genes involved in the metabolism of low-density lipoprotein (LDL), including the LDL receptor (LDLR), Apolipoprotein B (APOB), or Proprotein Convertase Subtilisin/Kexin Type 9 (PCSK9). FH causes increased LDL-cholesterol levels, leading to an increased risk for premature atherosclerotic cardiovascular disease.

This comprehensive review aims to discuss the progress of FH management, from classic statin therapy to relatively new therapies such as PCSK9 inhibitors and emerging gene-editing technologies like CRISPR. Furthermore, the article focuses on psychosocial aspects of adherence, such as patient beliefs, cultural influences, and healthcare access, and their impact on treatment outcomes. By examining these emerging treatment approaches, this review aims to create a broader understanding of FH management, focusing on better patient care and reducing the global burden of this condition.

## Introduction and background

Familial hypercholesterolemia (FH) is one of the most common monogenic disorders, affecting 1 in 250 individuals worldwide. The disorder results from mutations in genes responsible for critical pathways in lipid metabolism, such as low-density lipoprotein receptor (LDL-R), Apolipoprotein B (APOB), or proprotein convertase subtilisin/kexin type 9 (PCSK9), causing persistently elevated levels of low-density lipoprotein cholesterol (LDL-C) [1]. If left untreated, FH greatly increases the risk of atherosclerotic cardiovascular disease, which often manifests in early adulthood or even childhood. Despite significant advances in understanding its pathophysiology and treatment options, studies estimate that fewer than 10% of FH cases are diagnosed globally, and of those diagnosed, many remain undertreated [2].

Management of FH has evolved over decades from dietary interventions and statins to novel therapies involving PCSK9 inhibitors and gene-editing technologies. While these new developments hold promise for major reductions in LDL-C, optimal outcomes depend not just on the efficacy of these interventions but also on patients' adherence, which can be shaped by their cultural, socioeconomic, and educational backgrounds. Beliefs about the necessity, efficacy, and safety of therapies significantly influence adherence to lifelong treatment plans. For example, misconceptions about potential side effects or the perceived lack of immediate benefits may reduce adherence, leading to suboptimal clinical outcomes [3].

This review synthesizes the recent progress in FH management and examines the psychosocial dimensions of adherence among patients. We will explore how behavioral factors such as patient beliefs influence adherence and treatment efficacy by integrating existing evidence and highlighting practical approaches to addressing these barriers. By addressing both scientific and behavioral aspects of FH management, this review aims to provide a comprehensive perspective on improving care and reducing the global burden of this condition.

## Review

Current and emerging treatments for FH and its side effects

FH treatments have evolved significantly, with an increasing number of pharmacological and innovative approaches available for managing elevated LDL-C. However, these treatments are associated with varying degrees of side effects, long-term outcomes, and potential complications, particularly as newer therapies emerge. Statins are known for causing myopathy and elevations in liver enzymes such as alanine aminotransferase (ALT) and aspartate aminotransferase (AST). Ezetimibe commonly causes gastrointestinal symptoms, including diarrhea and abdominal pain. PCSK9 inhibitors, while generally well-tolerated, can cause injection site reactions and mild upper respiratory symptoms like nasopharyngitis. This review summarizes the current understanding of side effects and outcomes associated with established therapies and explores the potential risks of novel treatments [4].

Statins

Statins form the cornerstone of the management of FH through their inhibitory action on HMG-CoA reductase, which reduces hepatic cholesterol synthesis and upregulates LDL receptor activity. Although they are very effective in decreasing cardiovascular events, their side effects remain a concern for many patients. Myalgia, gastrointestinal disturbances, and mild increases in liver enzymes have been the most common adverse effects reported [5]. Rare but serious complications such as rhabdomyolysis and hepatotoxicity can occur, particularly at higher doses or in patients with predisposing factors [6]. Despite these risks, long-term studies such as the Scandinavian Simvastatin Survival Study (4S) demonstrate significant reductions in all-cause mortality, affirming their critical role in managing FH [7].

Ezetimibe

Ezetimibe is often used as an adjunct to statins, particularly in patients with insufficient LDL-C reduction or statin intolerance. By inhibiting cholesterol absorption in the intestine, ezetimibe offers an alternative mechanism of action. Adverse effects are generally mild, including diarrhea, fatigue, and abdominal pain [8]. When combined with statins, it provides additive LDL-C reduction and cardiovascular risk reduction, with a favorable long-term safety profile [9].

Proprotein Convertase Subtilisin/Kexin Type 9 (PCSK9) Inhibitors

PCSK9 Inhibitors, such as alirocumab and evolocumab, are monoclonal antibodies that increase the reutilization of LDL receptors [10]. These have been game-changing therapies in the management of FH and specifically in those with severe hypercholesterolemia or statin intolerance, injection site reactions and mild flu-like symptoms such as fatigue, muscle aches, and fever are common adverse effects, and neurocognitive impairment has rarely been reported. The FOURIER trial showed that evolocumab reduced major adverse cardiovascular events by 15%, including cardiovascular death, myocardial infarction (MI), and stroke. Injection site reactions occurred in 2.1% of patients on evolocumab compared to 1.6% in the placebo group. Neurocognitive events were reported in 1.6% of evolocumab users versus 1.5% in the placebo group, with no significant difference. These adverse effects were generally mild and transient, highlighting the favorable safety profile of PCSK9 inhibitors [10].

Emerging treatments: complications and unknowns

Inclisiran

The siRNA therapy inclisiran is taken up by hepatocytes and mediates the degradation of PCSK9 mRNA, thereby reducing its translation. This novel mechanism provides sustained LDL-C lowering with twice-yearly dosing. Although early trials report favorable safety profiles including minimal systemic side effects of injection site reactions and flu-like symptoms, long-term consequences of RNA interference are not well understood with regard to immune activation or unintended off-target effects [11].

Gene-Editing Technologies

Gene-editing therapies, especially those using CRISPR-Cas9, seek to correct pathogenic mutations in LDLR, PCSK9, or other FH-causing genes. While preclinical models have shown great promise, there are also significant risks with the therapies, including but not limited to off-target genetic modifications, insertional mutagenesis, and immune responses. The absence of human data on long-term exposure hinders proper understanding of their safety and efficacy in the clinical environment [12].

ANGPTL3 Inhibitors

Antisense oligonucleotides directed against ANGPTL3, as with evinacumab, offer a new pathway, independent of the LDL receptor, to lower LDL-C. Although very effective, these new therapies are also associated with hepatic enzyme elevations and injection site reactions. The involvement of ANGPTL3 in the broader metabolic regulation thus raises concerns regarding off-target effects, including glucose metabolism or dysfunction in adipose tissue, which will have to be further studied. This treatment regimen is associated with some common side effects. These include mild flu-like symptoms, such as nasopharyngitis, dizziness, and rhinorrhea, as well as nausea. Hepatic enzyme elevations have also been reported, although these are generally mild and temporary. While the drug is generally well-tolerated, these side effects, along with potential concerns about glucose metabolism and adipose tissue function, require further investigation [13].

The impact of coenzyme Q10 in FH treatment

Coenzyme Q10 is a lipid-soluble natural antioxidant component of every cell in the body and plays a crucial role in mitochondrial energy production. Interest in its use in the treatment of FH has been growing, especially since statin medications, prescribed to the majority of FH patients, have been associated with reduced levels of CoQ10. Statins act by inhibiting HMG-CoA reductase, which is also an enzyme involved in the synthesis of cholesterol and CoQ10, and this may promote symptoms such as muscle pain, fatigue, and weakness that have been associated with statin therapy. While all statins can potentially reduce CoQ10 levels, some statins, such as simvastatin and atorvastatin, are more likely to cause greater depletion than others. This can lead to a higher incidence of muscle-related side effects, such as myopathy or rhabdomyolysis, depending on the statin and the dose [14].

Research has documented that supplementation with CoQ10 can ease some of these side effects by restoring levels of CoQ10 and thus supporting mitochondrial function. Several studies have demonstrated an improvement in muscular function, a reduction in fatigue, and even an improvement in general quality of life in statin-treated patients receiving CoQ10 supplementation. In the case of FH patients, who often have to undergo prolonged therapy with statins, CoQ10 supplementation might prove to be a useful approach toward enhancing treatment tolerance and compliance, with a view to improving long-term outcomes [15].

In addition, CoQ10 has potential benefits other than those related to statin-induced side effects. As an antioxidant, it can help reduce oxidative stress and may be cardioprotective; thus, especially useful in patients with FH who have a high risk of cardiovascular events. Several studies have pointed out that CoQ10 could also be useful in improving endothelial function, an essential mechanism in the management of FH-related atherosclerosis [16]. While the therapeutic effects of CoQ10 are promising, larger studies are still required to fully establish its role in the treatment of FH and whether it can usefully complement lipid-lowering therapies in reducing cardiovascular risk.

Coenzyme Q10 Combined With Omega-3 Therapy in FH

The combination of coenzyme Q10 and omega-3 fatty acids might be a critical therapeutic option in the management of FH patients who experience statin-induced side effects or need additional cardiovascular protection. Coenzyme Q 10 And omega-3 fatty acids exert their actions through separate mechanisms, which might show some synergism that could further help in the management of FH, especially in reducing cardiovascular risk and improving patient adherence to long-term treatment regimens [17]. 

Coenzyme Q10 and Omega-3 Fatty Acids: Mechanism of Action

CoQ10 is a powerful antioxidant involved in mitochondrial energy production; its supplementation could mitigate the adverse effects of statins, such as muscle pain and general fatigue, commonly seen in FH patients. Omega-3 fatty acids mainly present in fish oils are known to possess anti-inflammatory properties with cardioprotection, including reduction of triglycerides, enhancement of endothelial function, and reduction in the risk of atherosclerosis-a major concern in FH patients because of their high cholesterol levels [18].

Combining CoQ10 with omega-3 therapy may be complementary in providing benefits to FH patients. While CoQ10 helps in maintaining mitochondrial function and reducing muscle discomfort associated with statin use, omega-3 fatty acids may add further cardiovascular protection by improving lipid profiles. Omega-3 fatty acids decrease triglyceride levels and might increase HDL-C, thereby contributing to an improved overall lipid balance in FH patients. Combination of such therapy with statins can further provide improved lipid profiles and hence can also counteract common side effects related to statin use [19]. Furthermore, both CoQ10 and omega-3 fatty acids have anti-inflammatory properties that may be particularly important in reducing the atherosclerotic inflammation characteristic of FH. CoQ10 has been identified as improving endothelial function, and omega-3s stabilizing plaques to reduce the possibility of rupture of plaques with subsequent cardiovascular events. Several studies have explored the effects of CoQ10 combined with omega-3 on different cardiovascular conditions. For example, a study on patients with hyperlipidemia demonstrated that CoQ10 supplementation improved muscle symptoms and omega-3 supplementation reduced triglyceride levels, indicating potential benefits when used together. However, while these results are promising, research specifically focused on FH is limited. Nevertheless, the combination holds significant potential for reducing cardiovascular risk and improving treatment adherence in FH patients who experience statin intolerance [20].

Implications and strategies for risk mitigation

Side effects and complications of current and novel treatments for FH emphasize the importance of an individual approach to management. The monitoring of biochemical markers, including liver enzymes and creatine kinase, is necessary for early detection of adverse events, especially in high-risk patients. In clinical practice, pharmacogenomic knowledge may further optimize treatment choice and dosing, especially regarding statins and novel treatments such as inclisiran [21]. Patient compliance remains a strong predictor of long-term results. Education and shared decision-making regarding side effects will improve adherence, while the use of digital health monitoring tools may facilitate early intervention for treatment complications [22]. Long-term safety studies for these emerging therapies should be a priority in future research, along with exploring potential synergies between traditional and novel approaches to optimize efficacy while minimizing risks. Tailored medicine and artificial intelligence have the potential to further develop risk stratification and personalize treatment plans to ensure efficacy and safety in the management of FH [23].

Clinical manifestations of FH and effect of comorbid conditions

Through a review of the patients record from the Eastern Danish Heart Registry and national administrative registries, a population of patients with MI was selected and referred for coronary angiography in the study. The study population was divided into two groups: "unlikely FH" and "possible FH". These patients with MI who had possible FH had similar levels of comorbidities and were treated more aggressively with cholesterol-lowering drugs compared with patients unlikely to have FH. Event rates of recurrent MI were higher in patients with possible FH than in those with unlikely FH. The study concluded that among patients with a history of MI, those with possible FH are at a higher risk of recurrent MI but similar mortality risk compared with patients with unlikely FH [24]. Many times, unusual findings that the patient may have been brought to the physician's attention by the patients or their relatives, and physical examination findings suggestive of the condition include arcus corneae, tendon xanthomas, xanthelasma, or tuberous xanthomas. Also, FH patients can present with premature coronary heart disease that raises suspicion for the condition. The presence of tendon xanthomas at any age can suggest FH. They are palpable nodular tendon irregularities on the extensor tendons of fingers or along the Achilles tendon. Arcus corneae in patients less than 45 years of age is also a possible indication of FH condition. They can easily be identified by directly inspecting the cornea. The peripheral cornea will appear opacified. Typically, as a milky white or blue gray color. Xanthoma is a deposit of cholesterol under the skin, on or around the upper eyelids; tuberous xanthoma is a large deposition of cholesterol commonly found over the joints. All of these above findings occurring in less than 25-year-old patients are indication of performing FH screening. Premature coronary heart disease refers to CHD occurring in men <55yrs and women <65 years. In these patients, any underlying cause including FH should be considered for their atherosclerotic disease. Lipid testing is optimally done via a fasting lipid panel. Other etiologies behind hyperlipidemia and contributors like diabetes, hypertension, or tobacco smoking have to be mentioned, too [25].

The role of tailored medicine

With the emergence of genomics and decoding of the human genomes, there has been growing enthusiasm for what is called personalized medicine, where management and treatment of patients are tailored to the patient individually [26]. In a study conducted and designed as a randomized and controlled trial and as a project called PRO-FIT, individuals with FH were assigned randomly to a control or to intervention group [27]. This project focuses on the evaluation and development of an innovative intervention, aiming to reduce cardiovascular diseases risk by promoting a healthy lifestyle between patients with FH. In the intervention group, participants received a personalized intervention which is a combination of web-based tailored lifestyle advice and also received personal counselling by a lifestyle coach. The control group received the usual care [27]. A study was conducted to evaluate the reach, the dose, and the fidelity of an individually tailored lifestyle intervention in people with FH, and the association between intervention dose and changes in LDL-Cholesterol (LDL-C) and multiple lifestyle behaviors in a 12-month follow-up. Associations were measured between intervention dose and change in lifestyle behaviors and the levels of LDL-C [28]. The LDL-C changes and lifestyle behaviors were associated with the dose of delivered PRO-FIT advice, face-to-face counseling, telephone booster calls, and the entire intervention-package. The entire delivery of the intervention-package resulted in non-significant improvements in LDL-C and lifestyle behaviors. In particular, the association between completion of different advice modules of PRO-FIT and LDL-C changes and related lifestyle behaviors were positive, but not statistically significant. Other studies also demonstrated this weak or no association in regard to lifestyle interventions from the web. In general, negative associations were found between the number of telephone booster calls and LDL-C level and the lifestyle behaviors but they were not statistically significant. It could be speculated that the fewer sessions performed, the more lifestyle behaviors improvements might already have been made, and no further session was necessary, since these participants were encouraged to plan telephone sessions by themselves, according to their need to have additional counselling [29].

Barriers to effective treatment

Analyzing a patient's adherence to the treatment may present barriers to the effective treatment of hypercholesterolemia. A study undertaken to identify the enablers and barriers in the treatment of FH analyzed for the presence of barriers to the treatment. In those individuals who knew how FH had affected their family members, it was the most common factor considered by the individuals when assessing their risk. Individuals whose family members were affected by FH and died early because of the condition or fell ill had a higher perceived risk, whereas those whose families were not affected by FH or whose family members were not affected by the consequences of the condition had a lower perception of risk. For most of them, the result of their risk analysis was that FH presented no serious current or future risk to their health. Such incongruence between perceived and objective risk has been identified as a barrier to treatment adherence [30]. Even when the risk to their health that FH posed was recognized, people generally thought there was the belief that they could modify their own individual risk. For them, they knew this implied active involvement in treatment and self-responsibility for their management of the condition. Treatment itself was perceived to be something effective as well as people having a perception about living with FH as being very treatable and controllable. In particular, medication was regarded by the participants as a necessity and an effective treatment modality. This belief that they were able to have successful self-care of their condition was found to facilitate treatment adherence. It was, however, perceived-the effectiveness of the medication-that lessened the importance attached to adhering to lifestyle treatment, and this prioritizing was found to represent a barrier for lifestyle treatment. Other barriers to treatment in FH patients included incorrect or inadequate knowledge of treatment advice, and concerns over the short and long-term use of lipid-lowering medication were identified as barriers to treatment [30].

The role of patient’s belief and medication adherence

Healthy Literacy and Information Source

The Internet Web provides easy access to a tremendous amount of information. It is increasingly used to locate information on specific topics. Among other things, a very popular information topic for Web searches is gathering information about a certain health condition. Individuals who seek medical information on the Web are by far nonprofessionals in a need for more or alternative information on the cure of a certain disease that should answer their health questions and allow them to make informed decisions. However, this is not always easy to achieve. While searching for medical information in the Web, people are often faced with conflicting evidence. Also, the publishing of the information on the Internet is not regulated, the low-quality health websites being regarded to be as common if not more frequent than the those providing high quality information [31]. Among such epistemic beliefs will be critical: "the confidence in one's ability to make good evaluations and to make good choices, which therefore helps individuals discern when websites present fact from opinion and bias". Epistemic beliefs denote beliefs about the nature of knowledge and also knowing the nature of this information. One study referred to them as a predictor of how people handle information from the Web, among other things, while pointing out that more advanced beliefs result in a more adequate handling of information from the Web. Because information on the Web not only offers access to new knowledge but also is often conflicting, it might provide a good environment to foster more advanced beliefs. One study specifically investigated the impact of receiving conflicting information on different levels of epistemic beliefs and also on decisions based on different kinds of information found on the Internet [31].

Psychosocial Factors Influencing Beliefs

A relation of more sophisticated epistemic beliefs with more knowledge indicates the impact of schooling on the development of epistemic beliefs. A Schommer study (1990) found that participants who completed more years of schooling were more liable to have the advancement of beliefs that knowledge is tentative, and development of epistemic beliefs across years of secondary school is related to learning during the secondary school years [32]. Cano also pointed out epistemic beliefs become more realistic and complex later as compared with the early year's secondary education [33]. On the other hand, there was an inverse relation between knowledge and epistemic beliefs. For instance, in a study on epistemic beliefs about physics between students, Köller, Baumert, and Neubrand (2000) found that the longer and more intensive the students learn about physics, the more they show dual views, and the more they thought that definitive truth could be obtained in the subject [34]. Therefore, it is possible to conclude that further knowledge gained influences epistemic beliefs, but the direction of change depends on the kind of knowledge acquired and also on people's previously held beliefs [35-37]. 

The Role of Healthcare Providers in Diagnosis and Screening

Using clinical diagnostic criteria and genetic testing, primary care doctors and lipid specialists play a crucial role in early detection. In order to identify impacted family members, which is essential for early intervention, healthcare providers should use cascade screening. Cascade screening helps identify at-risk family members of those with FH by testing first-degree relatives for high cholesterol levels and genetic mutations. Programs in the UK and Netherlands have been successful in finding undiagnosed FH cases and reducing long-term cardiovascular risks through early treatment. Gaps in knowledge, such as unfamiliarity with the latest treatments, can be addressed through targeted training programs, like the "FH Training Academy" in the Netherlands, which has improved the diagnosis and management of FH. There is a significant need for enhanced education and training programs for healthcare providers to improve their knowledge and awareness of FH [34-36], Cardiovascular nurses and other healthcare providers play a vital role in educating patients and their families about FH. Providers should adopt evidence-based, context-specific models of care that include patient-centered management, multidisciplinary teamwork, and high-quality clinical registries. Implementing integrated approaches within healthcare systems can improve the identification and management of FH, addressing care gaps and optimizing treatment [36].

Interventions to Improve Beliefs in Different Strategies' Adherence

Personalized lifestyle interventions based on the Transtheoretical Model of Health Behavioral Change have shown significant improvements in healthy lifestyle changes and treatment adherence. These interventions also resulted in reductions in body mass index, LDL-C, and blood pressure. Educating patients, their families, and healthcare providers about FH and the benefits of lipid-lowering treatments (LLT) is crucial. This includes addressing misunderstandings about the risks and benefits of treatment and providing standardized treatment guidelines. Providing patients with printed information, compliance check cards, and text message reminders has been effective in improving adherence to treatment and achieving target cholesterol levels [37]. Interventions for behavior change techniques (BCTs) can also be used to increase adherence to dietary and physical activity recommendations, interventions utilizing the Theoretical Domains Framework (TDF) and Behavior Change Wheel (BCW) have been developed. These consist of sections on problem-solving and family-based consultations that are followed by phone assistance [38-40]. Promoting healthy lifestyle practices and enhancing biological markers of cardiovascular disease risk have been successfully accomplished through the use of both web-based customized lifestyle advice and one-on-one lifestyle coach counseling. Customized interventions by general practitioners and community pharmacists have lowered cholesterol, encouraged healthier lifestyle choices, and greatly enhanced statin adherence [38]. It is crucial to create and test implementation plans in order to encourage the use of treatments for FH that are advised by guidelines. This entails determining the obstacles and enablers, as well as utilizing models such as the Practical, Robust Implementation and Sustainability Model to direct the creation and assessment of these tactics [39].

Strategies to Enhance Patient Communication and Empowerment

Cascade testing, or the stepwise and systematic screening of relatives at-risk in the family, is an effective way of identifying additional individuals with FH, since most individuals with the disease have an autosomal dominant form of the condition. However, cascade testing for FH is not routinely done specifically in the U.S. and the sharing risk information about FH and motivating family to test is left to the proband or first person diagnosed with FH. The probands report many challenges to communicating about FH with their family: difficulties in recalling complex risk information; geographical and emotional distance to relatives at-risk and inability to motivate them to pursue diagnosis and treatment. Probands could be given the concept of Dear Family Letters for relatives at risk, which try to aid family communication and cascade testing, but such passive approaches remain suboptimal in reaching a wanted goal. Comparatively, active means-like, direct contact of the physicians with the relatives-had higher rates of new relatives with FH identified per proband. Also, persons with FH expressed a wish to receive support from the clinicians themselves in communicating the health risk related to FH to their relatives. Other potential solutions to lighten the communication burden of probands and increase the uptake of cascade testing for FH include novel and active communication strategies, including digital tools and direct contact [40]. Such digital tools, like chatbots, could support patients through the provision of standardized medical information designed by clinicians at a user's own pace, thereby increasing access to genetic counseling and testing resources. These are digital agents that communicate in ways similar to human dialogue and have been implemented in healthcare systems for the delivery of genetic information. Another active strategy, with a higher likelihood of reducing proband burden and/or increasing cascade testing uptake, may be direct contact. Patient decision aids (PDAs) can support the process of shared decision making (SDM) and enable patients in making decisions that are deemed to be consistent with a person's goals and values. The purpose of this paper is to describe findings of a study designed to inform the development of a PDA for patients with FH through the use of provider perspectives of decision aid in FH using a qualitative inductive approach and focus group discussions of patients, providers, and genetic counselors. SDM can be created in the first instance by the role of decision aids. Physicians did mention, however, that decision aids, if used correctly, could free up more time to discuss decisions in consultations for SDM to take place. One physician also commented that though PDAs ideally help patients make decisions more consistent with their goals and values, such tools should not be persuasively used to impose clinicians' views that do not necessarily match the patients' preferences [41]. 

Discussion

Therapy for FH has greatly evolved and modernized over and above mainly pharmacological treatments. First, statins are the first-line treatments because of their established LDL cholesterol lowering and event reduction capability. However, their potential side effects, including myalgia and gastrointestinal disturbances, limit their use in some patients. Ezetimibe is given with statins, which result in further LDL-C lowering but is generally mild in adverse effects; however, the improved risk reduction would not be major. The great advancement is in the area of PCSK9 inhibitors like alirocumab and evolocumab, which can lead to tremendous LDL-C reductions, particularly for those in severe or intolerant statin conditions but these are quite costly and still need long-term safety and cognitive effect data [4-8]. The newest treatment such as inclisiran and gene-editing technologies like CRISPR-Cas9 show up novel ways to manage FH. Inclisiran, a small interfering RNA, has the promise of inducing prolonged LDL-C lowering with fewer doses each year that might really boost adherence and convenience; however, the therapy's long-term safety is still poorly understood. Gene-editing technologies could potentially provide curative forms of therapy for FH by directly removing genetic defects causing the disease, but ethical and safety concerns, such as off-target effects and unforeseen genetic complications, come with its application. Similarly, ANGPTL3 inhibitors open a new avenue for lowering LDL-C; however, we need more insight into their efficiency within longitudinal metabolic processes to avoid setting new health risks [11,12].

The psychosocial aspects of FH management are equally important. Beliefs and perceptions of the patients about the necessity, efficacy, and safety of treatments are crucial in adherence to the prescribed therapies. Even the best treatment becomes ineffective if a patient doesn't accept it because of widespread misconceptions or apprehension about taking medication for a long time. Improving patient education, creating a supportive healthcare environment through shared decision-making, and mobilizing digital health tools for monitoring adherence and providing encouragement are indeed important. By this, treatment outcomes should dramatically improve, and the patient will be engaged in his lifelong management plans [29,34-36].

Future directions and recommendations

Advances in the treatment of FH have significantly enhanced the management, but a number of important areas need further development. The inclusion of precision medicine, novel therapies, and a deeper understanding of patient beliefs will be critical in optimizing outcomes in patients with FH [42,43]. Precision medicine is an important future direction for further expansion. Genetic and pharmacogenomic profiling can help in personalizing treatments to specific patients to enhance efficacy and reduce adverse effects. Continued research is also needed in newer treatments, including gene-editing technologies and RNA-based treatments, to hone their safety, efficacy, and long-term consequences. Incorporation of digital health tools, including telemedicine and digital monitoring, may enhance adherence among patients and permit the observation of treatment effectiveness in real time. Moreover, health disparities in access to these advanced treatments play a critical role in improving the current global health inequities in managing FH. Clinical practice should be done in a multidisciplinary manner [43]. This would include genetic counselors, lipidologists, and psychologists who can address the medical and psychosocial needs of the patients with FH. Future research is also needed to emphasize long-term safety, especially for newer therapies, and to determine how patients' beliefs and behaviors about treatments affect adherence. Trials of comparative effectiveness will also be required to establish the proper therapy for subsets of patients with particular disease features. On a broader scale, public health initiatives should advocate for universal screening programs to ensure early diagnosis, while awareness campaigns can help improve patient understanding and reduce the stigma associated with cholesterol-lowering therapies. The future of FH management is moving toward precision medicine, expanded access to novel therapies, and behavioral aspects of patient care. All these areas will not only maximize treatment outcomes but also reduce global FH burden [44].

The integration of artificial intelligence and machine learning into tailored medicine promises to optimize FH care. Predictive models incorporating genetic, clinical, and lifestyle data can identify at-risk individuals earlier and refine treatment algorithms. Furthermore, advancements in gene-editing technologies, such as CRISPR-Cas9, hold potential for curative approaches by directly correcting pathogenic mutations. Collaborative research efforts are essential to translate these innovations into clinical practice, ensuring equitable access and minimizing disparities in care [45].

## Conclusions

Progress made in FH treatment has advanced significantly in managing this condition, although challenges remain. These include the incorporation of new therapies, patient-centeredness, and greater insight into the psychosocial processes influencing adherence. Future pathways must include precision medicine approaches, continuous research for further development of new treatments, and implementation of models for comprehensive care addressing both behavioral and medical conditions that characterize FH management. Ultimately, the education of the patients and SDM will contribute significantly to covering most of these aspects within a multidisciplinary approach including treatment outcomes and the long-term risks of this disorder.
